# Case Report: Pityriasis Rosea-Like Eruption Following COVID-19 Vaccination

**DOI:** 10.3389/fmed.2021.752443

**Published:** 2021-09-07

**Authors:** Kanchana Leerunyakul, Kallapan Pakornphadungsit, Poonkiat Suchonwanit

**Affiliations:** Division of Dermatology, Faculty of Medicine, Ramathibodi Hospital, Mahidol University, Bangkok, Thailand

**Keywords:** adverse effect, coronavirus, cutaneous eruptions, immunization, pityriasis rosea, SARS-CoV-2, vaccine, case report

## Abstract

Vaccination is one of the cornerstones in the efforts towards ending the coronavirus disease 2019 (COVID-19) pandemic. However, several adverse effects of COVID-19 vaccination have been identified. Pityriasis rosea (PR)-like eruption is a rare cutaneous complication of immunization. To the best of our knowledge, there have been no reports of PR-like eruptions following inoculation with Oxford/AstraZeneca ChAdOx1 nCoV-19 (AZD1222) vaccine. Here, we described a case of PR-like eruption that developed 14 days after Oxford/AstraZeneca vaccination in a 52-year-old Thai woman with glioblastoma. Treatment with topical 0.1% triamcinolone acetonide twice per day showed partial response after seven days. Despite this rare complication, our report highlights that the presence of PR-like eruption is not a contraindication for subsequent vaccinations.

## Introduction

Pityriasis rosea (PR)-like eruption is a cutaneous complication associated with several medications and vaccinations. Due to the rising incidence of coronavirus disease 2019 (COVID-19) worldwide, mass vaccination has been the cornerstone in preventing viral spread and decreasing morbidity and mortality. However, adverse effects to COVID-19 vaccines, particularly cutaneous reactions, have been reported. Here, we describe a case of PR-like eruption following ChAdOx1 nCoV-19 (AZD1222) vaccination in a Thai patient with glioblastoma.

## Case Description

**Figure 1:**
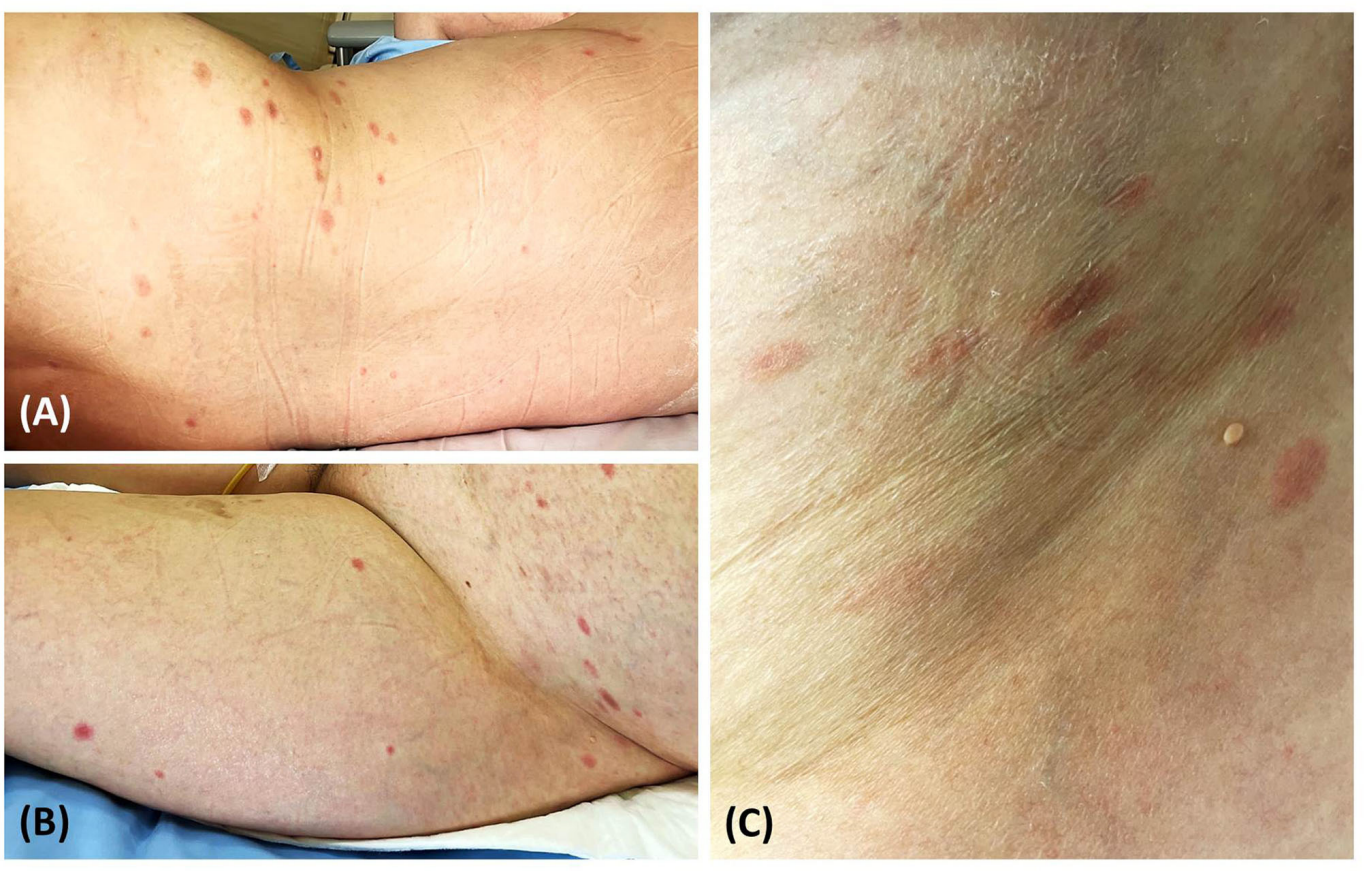
Pityriasis rosea-like eruption presenting as multiple discrete erythematous oval patches and plaques on the back **(A)** lower abdomen, and bilateral upper thighs **(B)**; lesions distributed along cleavage lines without prominent scales **(C)**.

**Figure 2:**
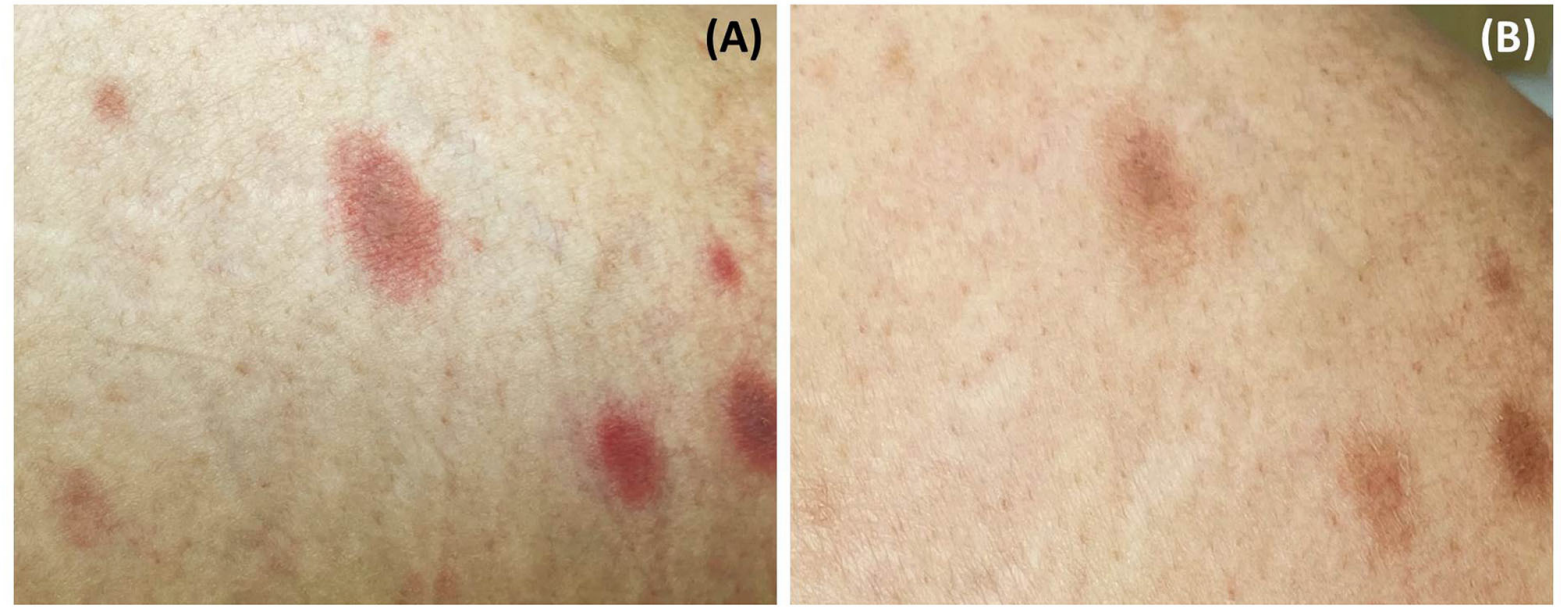
Central dusky-red erythematous on some plaques **(A)**; almost all lesions fading with associated post-inflammatory hyperpigmentation after treatment **(B)**.

A 52-year-old Thai woman with a one-year history of recurrent glioblastoma was admitted to our hospital for a planned targeted therapy. Following the national COVID-19 vaccination guidelines, she received a shot of ChAdOx1 nCoV-19 (AZD1222) vaccine before the therapy. However, after 14 days, she developed multiple discrete blanching erythematous oval patches and plaques on the lower abdomen, back, and bilateral upper thighs (Figures 1A,B). Physical examination revealed that lesions were distributed along cleavage lines, similar to PR, without herald patch or prominent scales (Figure 1C). Moreover, central dusky-red erythematous was observed on some plaques (Figure 2A). Few brownish patches also appeared, suggestive of post-inflammatory hyperpigmentation. Unfortunately, due to her deteriorating neurologic status, her associated symptoms were not evaluated properly. Nevertheless, she did not present with fever or respiratory tract symptoms. In addition to COVID-19 vaccination, she was currently taking medications (i.e., levetiracetam and thyroxin) for glioblastoma, and the two drugs had been prescribed for approximately nine months.

**Figure 3:**
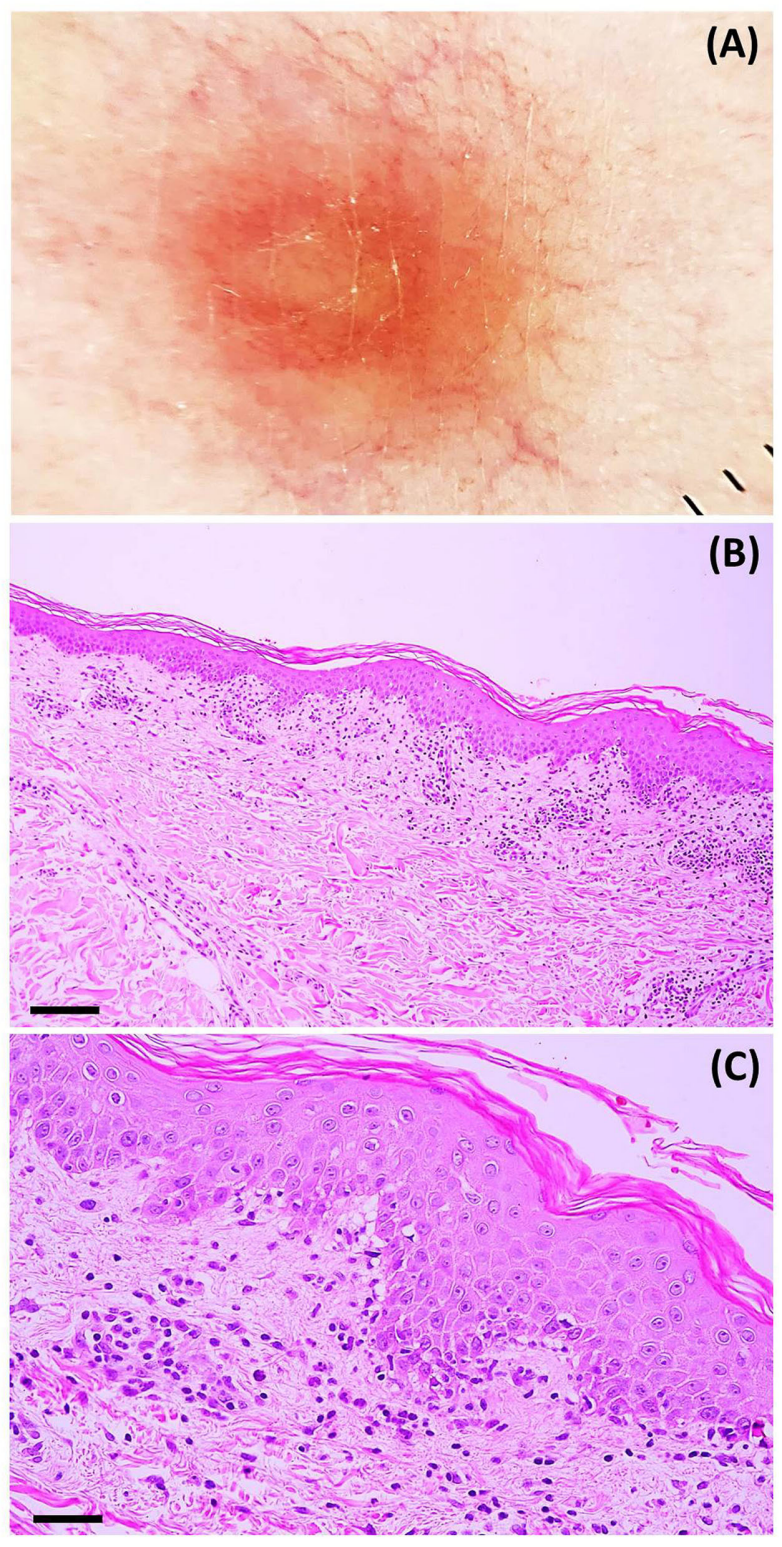
**(A)** Dermoscopic features: dotted and linear vessels forming a network on a yellowish background with some whitish peripheral scales (original magnification x20). Histopathologic features: **(B)** microscopic image at a low power field showing psoriasiform epidermal hyperplasia, perivascular infiltration, and interface dermatitis (hematoxylin-eosin; scale bar represents 100 μm); **(C)** microscopic image at a high power field showing focal spongiotic dermatitis, interface dermatitis, and perivascular infiltration of lymphocytes and eosinophils (hematoxylin-eosin; scale bar represents 40 μm).

Laboratory investigation results (i.e., complete blood count, blood chemistry, C-reactive protein level, erythrocyte sedimentation rate, serological test for syphilis, plasma polymerase chain reaction (PCR) for human herpesvirus (HHV)-6 and HHV-7, and reverse transcription-PCR for COVID-19) were unremarkable. Dermoscopy showed dotted and linear vessels forming a network on a yellowish background, with some whitish peripheral scales (Figure 3A). Histopathologic examination revealed epidermal focal spongiosis, interface dermatitis with necrotic keratinocytes, perivascular lymphocytic infiltration, and few eosinophilic infiltrations in the superficial dermis (Figures 3B,C). These findings were consistent with a diagnosis of PR-like eruption. Due to the temporal relationship between vaccination and onset of rash, COVID-19 vaccination was the most likely etiology. Treatment with topical 0.1% triamcinolone acetonide twice daily showed partial response after seven days. Moreover, almost all lesions faded with associated post-inflammatory hyperpigmentation (Figure 2B).

## Discussion

PR is a common self-limited papulosquamous eruption typically presenting with a large solitary pink-colored patch or plaque (i.e., herald patch), followed by secondary eruptions of smaller erythematous patches or plaques with associated peripheral collarette scaling distributed along the cleavage lines of the trunk, forming a Christmas tree pattern (5). However, in cases of atypical PR, it may present with erythema multiforme-like lesions and purpura (5).

Although the exact pathogenesis of PR remains unknown, the endogenous systemic reactivation of HHV (i.e., type 6 and 7) in the skin has been implicated as a precipitating factor. Among the common cutaneous manifestations of COVID-19 (e.g., morbilliform eruptions, chilblains, and varicella-like vesicles), PR and PR-like eruptions have rarely been described (6, 7). The mechanisms behind these COVID-19-related symptoms remain unclear. Some studies have identified severe acute respiratory syndrome coronavirus 2 (SARS-CoV-2) spike proteins on endothelial cells and lymphocytes from PR-like lesions, consistent with direct viral invasion (3, 4). Moreover, COVID-19 may solely induce human herpesvirus reactivation through the immunomodulatory ability of SARS-CoV-2 to reactivate latent viruses (8, 9).

Drug-induced PR-like eruption accounts for 2% of all cutaneous adverse drug reactions (10). Common causes of drug-induced PR-like eruptions include angiotensin-converting enzyme inhibitors, non-steroidal anti-inflammatory drugs, and gold. It often presents with diffuse and confluent severely pruritic dusky-red erythematous lesions in the absence of herald patch (11). Involvement of the oral mucosa has also been reported in as many as 50% of all cases (11). Although histopathologic characteristics of PR are also observed in PR-like lesions, the presence of dyskeratotic keratinocytes, interface dermatitis, and eosinophilic infiltration in the dermis may be more prominent in PR-like eruptions (11). Moreover, while dermoscopy may facilitate the diagnosis of PR, its utility for atypical PR or PR-like lesions is still limited.

Vaccinations have been identified to rarely cause several cutaneous reactions. The most commonly inoculated vaccines include those against smallpox, followed by tuberculosis, poliomyelitis, influenza, and human papillomavirus virus (12, 13). Among patients who received COVID-19 vaccines, PR-like eruptions have been reported following the first (*n* = 1) or second dose (*n* = 2) of BNT162b2 mRNA-based vaccines (3, 4). In some cases, central hyperpigmentation was identified. Nevertheless, the incidence of skin reactions after mRNA vaccinations was low. In a registry-based study, PR-like eruption was observed in only 1–2% of patients (14).

To the best of our knowledge, only one case of ChAdOx1 nCoV-19 vaccine-induced PR-like eruption has been reported (2). The patient developed papulovesicular lesions in the crease lines of the trunk and extremities, four days after inoculation with a recombinant coronavirus vaccine (Covishield™, India). Among patients who received inactivated vaccines, another case of PR-like eruption was reported four days after the first dose of CoronaVac® (SinoVac) (1). Here, we report the first case of PR-like eruption following ChAdOx1 nCoV-19 (Oxford/AstraZeneca) vaccination. Table 1 summarizes all reported cases of PR-like eruption following COVID-19 vaccination. Although the exact pathophysiologic mechanisms of PR after vaccination remain unclear, immune responses to COVID-19 vaccines and disruption of T-cell mediated control of latent infections were implicated (12, 15–18). Other plausible theories include molecular mimicry with viral epitopes and type IV delayed hypersensitivity reaction (12).

**Table 1:** Clinical manifestations and histopathologic findings of reported patients with pityriasis-rosea like eruptions following COVID-19 vaccination.

**Author, years**	**Age**	**Sex**	**Comorbidity**	**Type of vaccines**	**Dose**	**Days of onset after vaccination**	**Days of total course**	**Clinical presentation**	**Histopathology**	**Treatment**
Akdas et al., (1)	45	F	None	Inactivated CoronaVac	1^st^	4	7	- Multiple round, salmon-colored plaques on trunk and extremities, resembling a Christmas tree distribution	- Focal parakeratosis - Exocytosis of lymphocytes - Spongiosis - Extravasated red blood cells in the dermis	- Mid-potency topical corticosteroid cream
Adya et al., (2)	21	M	None	ChAdOx1 nCoV- 19, Covishield™	1^st^	4	N/A	- Papulovesicular eruptions on trunk and extremities along skin cleavage line - A targetoid aspect with central hemorrhagic crust on some lesion**s**	- Spongiosis - Perivascular lymphocytic infiltrate - Extravasated red blood cells in the dermis	N/A
Abdullah et al., (3)	40	M	None	BNT162b2 mRNA	2^nd^	7	21	- Disseminated erythematous scaly patches on trunk and extremities - Herald patch on back	N/A	- Triamcinolone acetonide cream 0.1%
Cyrenne et al., (4)	20s	F	Alopecia areata	BNT162b2 mRNA	1^st^ and 2^nd^	2	14	- Large, red-to-tan, scaly plaques at the inoculation site followed by eruptions on the trunk	- Parakeratosis - Minimal acanthosis and spongiosis - Interface changes - Scattered dyskeratotic keratinocytes	- Topical corticosteroids
	40s	M	None	BNT162b2 mRNA	2^nd^	21	21	- Pruritic, red-to-tan, small oval plaques with peripheral scales on the trunk and proximal extremities - Herald patch on left axilla	N/A	- Doxycycline - Bilastine
Present case	52	F	Recurrent glioblastoma	ChAdOx1 nCoV-19, Oxford/AstraZeneca	1^st^	14	7	- Multiple erythematous to dusky oval patches and plaques on the trunk and upper legs	- Epidermal focal spongiosis - Interface changes and necrotic keratinocytes - Superficial lymphocytic infiltrate	- Triamcinolone acetonide cream 0.1%

*F, female; M, male; N/A, not available*.

In conclusion, we have described the first case of PR-like eruption following ChAdOx1 nCoV-19 (Oxford/AstraZeneca) vaccination. Fortunately, PR-like eruption is responsive to topical corticosteroids without any systemic adverse effects. Despite this rare complication, our report highlights that the presence of PR-like eruption is not a contraindication for subsequent vaccinations. With COVID-19 vaccines being authorized for emergency use, monitoring for potential adverse effects (e.g., cutaneous reactions) is necessary.

## Data Availability Statement

The original contributions presented in the study are included in the article/supplementary material, further inquiries can be directed to the corresponding author/s.

## Ethics Statement

Ethical review and approval was not required for the study on human participants in accordance with the local legislation and institutional requirements. The patients/participants provided their written informed consent to participate in this study. Written informed consent was obtained from the individual(s) for the publication of any potentially identifiable images or data included in this article.

## Author Contributions

KL drafted the original manuscript. KL and KP collated resources. PS conceptualized the case report and revised the manuscript. All authors approved the final manuscript as submitted and agree to be accountable for all aspects of the work.

## Conflict of Interest

The authors declare that the research was conducted in the absence of any commercial or financial relationships that could be construed as a potential conflict of interest.

## Publisher's Note

All claims expressed in this article are solely those of the authors and do not necessarily represent those of their affiliated organizations, or those of the publisher, the editors and the reviewers. Any product that may be evaluated in this article, or claim that may be made by its manufacturer, is not guaranteed or endorsed by the publisher.
